# Chitosan-Lemongrass Essential Oil on Paperboard for Active Food Packaging Applications

**DOI:** 10.3390/polym17040473

**Published:** 2025-02-11

**Authors:** Mariangela de Fátima Silva, Julia Vaz Ernesto, Alessandra Rigo Rinaldi, Ana Paula Reis Noletto, Patricia Santos Lopes, Rosemary Aparecida de Carvalho, Vitor Augusto dos Santos Garcia, Cristiana Maria Pedroso Yoshida

**Affiliations:** 1 Institute of Environmental, Chemical, and Pharmaceutical Sciences, UNIFESP—Federal University of São Paulo, São Paulo 04021-001, SP, Brazil; 2 ITAL—Institute of Food Technology, Campinas 13070-178, SP, Brazil; 3 Faculty of Animal Science and Food Engineering, USP—University of São Paulo, São Paulo 05508-220, SP, Brazil; 4 Faculty of Agricultural Sciences, UNESP—São Paulo State University, Av. Universitária, 3780, Botucatu 18610-034, SP, Brazil

**Keywords:** coating paperboard, chitosan, emulsion, lemongrass, mechanical properties

## Abstract

An active film-forming solution of chitosan and lemongrass essential oil was applied as a coating on paperboard, forming an alternative and sustainable packaging material for food applications. The active paper-film systems were characterized by color parameters, thermogravimetric analyses, contact angles, Fourier transform infrared spectroscopy, X-ray diffraction, mechanical properties, and cytotoxicity. The active coated paperboard was homogeneous and yellowish in appearance. The tensile strength (transverse and longitudinal directions) was directly influenced by increasing the number of layers of the chitosan-lemongrass active coating. The oil concentration significantly affected the Taber stiffness (transverse direction). The active coatings with higher concentrations of lemongrass essential oil altered the thermal stability of the coated paperboard. The contact angle values were characteristic of hydrophobic materials. The coated systems presented three characteristic peaks in the X-ray diffraction analysis—2θ = 16.5°, 22.9°, and 29.8°—and an amorphous halo at 18.9°. The cytotoxicity analysis of the active material (1:40:5) indicated potential reductions in the lemongrass essential oil content to maintain cell viability while ensuring insecticidal efficacy, supporting its safe use as food-contact active packaging. In this way, the active packaging system based on a chitosan coating containing lemongrass essential oil on paperboard could be a type of efficient active material packaging which is safe in contact with food and sustainable for the environment.

## 1. Introduction

Adequate food packaging could significantly reduce global food waste by protecting it from pathogenic microorganisms, insect attacks, mechanical damage, and ultraviolet radiation [1]. Packaging material is important for food industries serving convenience foods, such as fast foods, ready meals, on-the-go beverages, and snacks [2]. Synthetic polymer packaging material persist in nature, leading to waste accumulation and microplastic formation, causing water, soil, and food pollution, and even being found in living organisms [3]. Environmental and health concerns have increased the demand for eco-friendly natural polymer materials to partially replace petroleum-based synthetic materials in the packaging sector [4]. Some countries have already started reducing the use of polymers which are difficult to biodegrade in single-use packaging.

Paper-based packaging contains the world’s most abundant and natural carbohydrate–cellulose, a biodegradable, low-cost, renewable, recyclable, and nontoxic resource [5]. Demand for sustainable paper packaging is increasing due to its excellent mechanical properties. However, the gas, water vapor, and grease permeabilities are generally extremely high for food product applications [6,7]. Coated paper is a technique for improving paper’s physical properties, appearance, or printability using synthetic polymers, perfluoroalkyl substances, wax, and other materials, compromising paper’s biodegradability and recyclability attributes. Biodegradable polymers derived from renewable sources have been applied to improve the performance of paper-based materials [8].

Polysaccharides have been studied as renewable and biodegradable food packaging materials. Chitosan is a linear polysaccharide resultant of the deacetylation of chitin present in insects and crustacean shells [9,10] and is environmentally friendly due to its biodegradability and low toxicity [11]. Chitosan comprises randomly distributed β-(1-4)-linked D-glucosamine (deacetylated unit) and N-acetyl-D-glucosamine. This molecular structure is similar to cellulose, but in chitosan, C-2 hydroxyl groups are replaced by acetamido groups [12]. Good adhesion between a chitosan coating and a cellulose-based substrate makes chitosan an attractive polymer for the barrier coating of cellulose-based materials for food packaging [13]. Chitosan coatings exhibit oxygen barrier and grease resistance [14]. However, due to its hydrophilic nature, adding hydrophobic compounds can improve chitosan’s water vapor barrier properties [15].

A water-resistant food contact material was developed by applying three layers of chitosan to the cellulose paper surface, as observed by the decrease in the contact angle of the coated surface compared to an uncoated sample [16]. Applying a filmogenic chitosan-oil solution as a coating on a paperboard surface can improve the functional performance of the packaging [17].

Active packaging technologies have potential benefits in food applications, as defined by innovative concepts in materials, to maintain or prolong the shelf-life, safety, quality, and integrity of food [18]. The demand for natural active substances is increasing, such as plant essential oil as a natural preservative due to its biological action in small concentrations. Incorporating essential oils as a natural bioactive compound in packaging has been presented with excellent bacteriostatic, antioxidant, and insect repellency activities, among others [19]. As essential oils are considered safe by the Food and Drug Administration (FDA), their compounds are a potential and interesting alternative to antibiotics. Furthermore, incorporating essential oils into food packaging can protect food and prevent degradation [1]. Most essential oils are characterized by a repellent effect on many insects, being potential options for producing active food packaging. However, they are highly unstable due to their volatility, which can be stabilized when incorporated into natural matrices [20].

Lemongrass essential oil is derived from *Cymbopogon citratus*, presenting excellent antimicrobial, anti-inflammatory, and antioxidant properties. The isolated lemongrass chemical classes are tannins, sterols, terpenoids, phenols, ketones, flavonoids, and sugars [21]. Terpenoids are of greater interest, as the essential oil of *C. citratus* consists mainly of the citral compound, which represents 84% of it by weight [22]. Citral (3,7-dimethyl-2,6-octadienal) is the name given to a natural mixture of two isomeric acyclic monoterpene aldehydes—geranial (trans-citral, citral A) and neral (*cis*-citral, citral B)—in addition to geranilic olefins, geranyl acetate, and myrcene [23]. Lemongrass incorporated into chitosan coating paperboard provided an anti-insect packaging material against weevils in wheat grains and pasta packages [19].

Chitosan coatings have been successfully applied to paper surfaces, enhancing their barrier properties for food packaging. Combining chitosan with lipids, such as beeswax or carnauba wax, has been proven effective in further improving water vapor resistance and moisture protection while maintaining the mechanical integrity of the paper substrate [24,25]. Preliminary tests using lemongrass essential oil as an active agent demonstrated excellent resistance to grease, water, air, and microbial contamination, with potential applications for extending the shelf lives of grain-based food products while providing active insect protection [19]. These advancements underscore the potential of sustainable, biodegradable, and multifunctional packaging solutions for the food industry.

This work aimed to characterize the packaging properties and cytotoxicity of active coated paperboard based on a lemongrass-chitosan coating to verify its potential application as a sustainable and alternative food packaging material, evaluating its mechanical, thermal, and surface properties as well as its safety for food contact.

## 2. Materials and Methods

### 2.1. Materials

The paperboard was 210 × 297 mm with a grammage of 240 g/m^2^ and thickness of 0.385 mm from Suzano Papel and Celulose SA in Brazil. Chitosan ChitoClear (from the shrimp species *Pandalus borealis*) was obtained from Primex^®^ Iceland (Siglufjörður, NE, Islândia), with a degree of deacetylation 95% e Mw = 1.47 × 105 gmol^−1^. Acetic acid PA (99.8%) was acquired from Synth^®^ (Diadema, SP, Brazil). Lemongrass essential oil (*C. citratus*) was obtained from steam distillation leaves (Quinarí^®^, Ponta Grossa, PR, Brazil).

### 2.2. Production of Paperboard

For the active coating filmogenic solution and coating procedure. chitosan filmogenic suspensions were prepared in an aqueous acetic solution added stoichiometrically and kept under continuous agitation for 1 h. The amount of acetic acid was calculated from the weight of the sample, considering the degree of acetylation (5%) and the weight of chitosan, to achieve protonation of all NH_2_ sites [15]. The lemongrass essential oil was added to the chitosan solution (20%, 30%, and 40% *w*/*v*) by emulsion under rigorous homogenization (Ultra-Turrax IKA T25-Digital, Staufen, Germany) at 20,000 rpm for 10 min, resulting in a film-forming active solution. Paperboard previously stored at 25 ± 2 °C, 50 ± 2 rh was coated with the film-forming solution using an automatic spreader (Zehntner^®^, Sissach, BL, Switzerland) at a thickness of 100 µm and speed of 10 mm/s. The samples were dried in an air-forced circulation drying oven (Marconi MA 035/100, Piracicaba, Brazil) at 120 °C for 90 s, resulting in an active paper-film system.

### 2.3. Experimental Design

The chitosan (C_C_), lemongrass essential oil (C_L_) concentrations, and number of layers (N_L_) of the coating are defined under Experimental Design 2^3^ (Table 1).

### 2.4. Analysis

Active paper-film systems were previously pre-conditioned at 25 °C and 50 ± 2% RH following ASTM D 685–93 [26]. Uncoated paperboard was used as a control sample.

#### 2.4.1. Color Parameters

The color parameters were determined on the face of the coating at 5 random points from 3 samples, totaling 15 repetitions, using a colorimeter (HunterLab^®^, Miniscan XE plus, Reston, VA, USA). Calibration of the equipment was performed using standard black and white plates. The color parameters were established using the CIE L*a*b* system. The L* parameter represents the lightness of colors (black = 0, white = 100), chroma a* represents greenness or redness (green = negative a*, red = positive a*), chroma b* represents blueness or yellowness (blue = negative b*, yellow = positive b*), and the values of the parameters a* and b* were converted to polar Hue coordinates (Hab = arctg (b*/a*)) [27].

#### 2.4.2. Contact Angle

The contact angle (CA) was obtained with pure water using a tensiometer (OCA 20^®^, DataPhysics Instruments, Filderstadt, BW, Germany) according to ASTM D724-99 [28]. Samples (2.0 × 2.0 cm) were fixed to the base of the equipment, and each drop was deposited with the rotating drop method (volume 15 µL) to control the volume and speed of the released drops. The equipment captured and calculated the contact angle using Attension Theta software (version 4.1.9.8) after 10 s of stabilization on the surface of the coated paperboard. A total of 20 repetitions were evaluated for each trial.

#### 2.4.3. Thermal Analysis: TG/DTG and DSC

The active paper-film systems and uncoated paperboard were analyzed in TG/DSC simultaneous mode equipment (NETZSCH^®^, STA F3 449, Jupiter, Selb, PY, Germany). The samples (10–20 mg) were packed in hermetically sealed aluminum capsules and subjected to a heating rate of 25–800 °C (10 K/min/800 °C) in an inert atmosphere [29]. The equipment’s software (https://analyzing-testing.netzsch.com/en/landingpages/software-and-dsc, accessed on 6 February 2025, NETZSCH^®^, Proteus Thermal Analysis, Selb, PY, Germany) was used for data processing and graphing.

#### 2.4.4. Fourier Transform Infrared Spectroscopy (FTIR)

The active paper-film samples and uncoated paperboard were analyzed using a Spectrum spectrophotometer (Perkin Elmer^®^, Waltham, MA, USA) with a universal attenuator of total reflectance (AURT) accessory at room temperature. The materials were placed directly over the sample compartment, and scans were carried out in the spectral range from 4000 to 450 cm^−1^ with a resolution of 4 cm^−1^ to identify the samples’ functional groups. The data obtained were plotted using Origin 2019 software.

#### 2.4.5. X-Ray Diffraction

X-ray diffraction data were collected using a diffractometer (Philips^®^, X’pert MRD, Amsterdam, NH, The Netherlands) equipped with a 0.15406 nm K Alpha reaction copper source. The measurements were performed using Bragg-Brentano geometry in the range from 5° to 60° [30]. The data were plotted using Origin^®^ 2019 (Northampton, MA, USA).

#### 2.4.6. Tensile Properties

The tensile strength and elongation at break of uncoated and coated paperboard were determined by the ASTM D828-16e1 tensile test [31] using a universal testing machine (Instron^®^ series 5900, Norwood, MA, USA) and a load of 1 KN. The specimens (180 mm × 15 mm) were cut using a guillotine and fixed to the equipment. The initial separation distance between the claws was maintained at 150 mm, and the speed of the initial test was 20 mm/min. The test room was maintained at 23 ± 2° C with a relative humidity of 50 ± 3%. For each test, 10 specimens were analyzed in the longitudinal direction of the fibers, and 10 specimens were analyzed in the transversal direction of the fibers. The tensile strength (MPa) and elongation at break (%) were calculated using the Bluehill^®^ software Bluehill 2 (Norwood, MA, USA) of the traction equipment.

#### 2.4.7. Taber Stiffness

According to ASTM D5342-97 [32], the uncoated and coated paperboard samples were cut to dimensions of 38.1 × 70.0 mm in the machine direction (MD) and the cross-machine direction (CD) using a guillotine (Regmed, São Paulo, SP, Brazil). The sample’s resistance to flexion at an angle of 15° was verified using a rigidity meter (Regmed^®^, model RI 5000, São Paulo, SP, Brazil). The results were expressed in mN with a 500 U counterweight for scale adjustment, with 10 repetitions for each direction.

#### 2.4.8. Direct and Indirect Cytotoxicity of the Active Film-Paper System

The International Organization for Standardization’s ISO 10993-5 [33] and 10993-12 (ISO, 2012) [34] standards were applied for this test, whose purpose is the biological evaluation of medical application devices in direct contact with skin or ingestion. Despite the high sensitivity, the analysis method adopted was an extrapolation of “in vitro” tests of cell viability of the active material. Fibroblast BALB/3T3 clone A31 (ATCC^®^ CCL-163™, Manassas, VA, USA) cells were grown in culture flasks with Dulbecco’s modified eagle medium (DMEM) supplemented with 10% (*v*/*v*) FBS, 1% (*v*/*v*) L-glutamine, and 1% (*v*/*v*) antibiotic solution (10,000 UI/mL penicillin, 10 mg/mL streptomycin, and 1 mg/mL amphotericin B). The cells were incubated at 37 °C in a 5% CO_2_ atmosphere with controlled humidity. The culture medium was changed every 2–3 days. After achieving 80–90% confluence, the cells were detached with 0.25% trypsin-EDTA solution. All experiments were performed using cells between passages 13 and 14. All of the reagents for cell culture were purchased from Vitrocell^®^ (Campinas, SP, Brazil).

**Figure 1:**
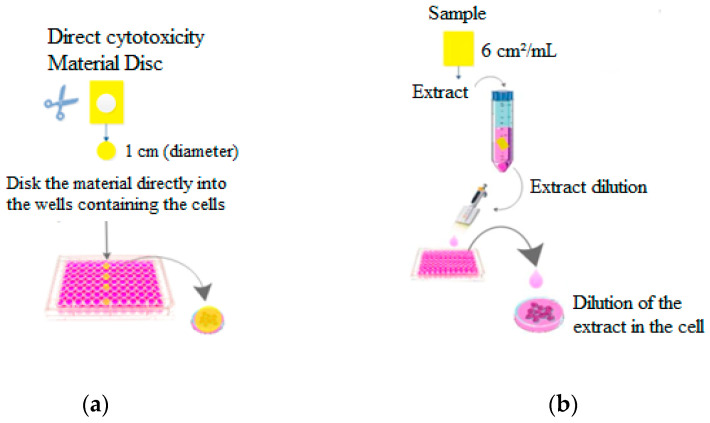
Scheme of cytotoxicity assay of the active chitosan-lemongrass paperboard systems: (**a**) direct and (**b**) indirect.

In the direct cytotoxicity method, discs of the active material, uncoated paperboard, and paperboard coated with chitosan (1 cm in diameter) were placed with the coating side in contact with the cultured cells and medium culture in the wells without propylene glycol (Figure 1a). In indirect cytotoxicity (Figure 1b), extracts were obtained from the samples (6 cm^2^/mL) of paperboard materials with D10 medium culture and propylene glycol for maximum extraction of oil and other components.

The lemongrass concentration (C_L_) in the active film-paperboard systems was estimated. Eight serial dilutions of the extracts were produced to verify cell viability (75–0.59 mg/mL). Results were obtained from the graphical assessment of cell viability.

### 2.5. Statistical Analysis

Statistical analyses were carried out using Statistica^®^ software 12.7 (Statistica^®^, Tulsa, OK, USA). Differences between the averages were identified via ANOVA and Tukey’s test (*p* < 0.05).

## 3. Results

### 3.1. Color Parameters

The chitosan-lemongrass essential oil coatings showed good adhesion and compatibility on the paperboard surface, as no delamination of the layers was observed after vigorous handling. This was also observed by Gällstedt et al. [35], who evaluated different film-forming solutions of natural polymers, including chitosan-coated paperboard. Good compatibility between the chitosan solution and cellulosic paperboard fibers was verified without coating delamination [16].

**Figure 2:**
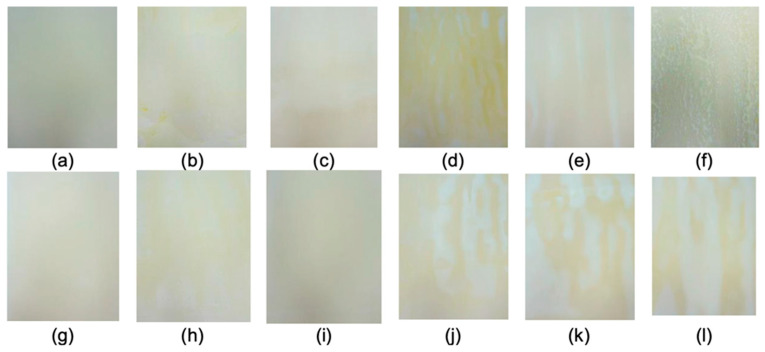
Active film-paperboard systems coated with different film-forming solutions according to the experimental plan (C_C_:C_L_:N_L_): (**a**) control (uncoated paperboard); (**b**) 3:40:5; (**c**) 3:40:1; (**d**) 3:20:5; (**e**) 3:20:1; (**f**) 1:40:5; (**g**) 1:40:1; (**h**) 1:20:5; (**i**) 1:20:1; (**j**) 2:30:3; (**k**) 2:30:3; and (**l**) 2:30:3.

Figure 2 presents the final appearance of the active film-paperboard systems coated with different formulations according to the experimental design. After drying, the coatings with one layer (1C) showed homogeneity. When applying three and five layers, depending on the amount of chitosan and lemongrass essential oil, heterogeneous areas were observed, which were associated with the filling of cellulose interfibrillar spaces and the formation of film-forming solution concentrates on the surface of the paper. When increasing the C_C_ concentration, the coated papers were characterized by greater handling resistance.

Color appearance is important for the consumer’s general appearance and acceptance of the packaging [9]. The color parameters L*, a*, and b* of the active film-paperboard systems were measured (Table 2).

For comparison, the color parameters of the control paper (uncoated) were measured (L* = 80.43 ± 0.37; b*= 16.79 ± 0.11; and a* = 3.29 ± 0.02). The parameter L* increased in all active film-paperboard systems compared with the control. The independent variables had no statistically significant effect on the L* values. Test 1 (maximum C_C_ and C_L_ and five coating layers) presented the highest L* value. The a* parameter was reduced compared with the control (3.29 ± 0.02) in all treatments. A positive and statistically significant effect on the a* parameter in the interaction of C_L_ and N_L_ was observed in the active film-paperboard systems. The b* parameter (values greater than zero tend to be yellow) showed higher values in the 3:40:5, 3:20:5, and 1:40:5 (C_C_:C_L_:N_L_) materials than the control (16.79 ± 0.11). The increased C_L_ in the emulsion coating provided a more yellow color. The chitosan solution at 3% (*w*/*v*) was characterized by a more yellow color than the C_C_ = 2% and 1% (*w*/*v*) solutions, which then contributed to higher values for b*. The responses had no statistical significance to the b* means. The values of the a* and b* parameters were converted into polar coordinates. The hue angle (Hab) indicated yellow tone values for all of the coated paperboard. When increasing the chitosan concentration and adding layers to the paperboard surface, the yellow color increased. The Hab values were higher for the five-layer treatments compared with the five-layer and three-layer treatments, represented by the positive and significant effect of N_L_ on the Hue angle response (i.e., increasing from −1 (one layer) to +1 (five layers)). The coated paperboard appeared more yellow, with an increase in the Hab of approximately 6.34°. The C_C_ also had a positive and significant effect on the Hab, increasing from −1 (1%) to +1 (3%), while the coated paperboard tended to have a more yellow coloration of about 3.37°.

### 3.2. Contact Angle (CA)

**Figure 3:**
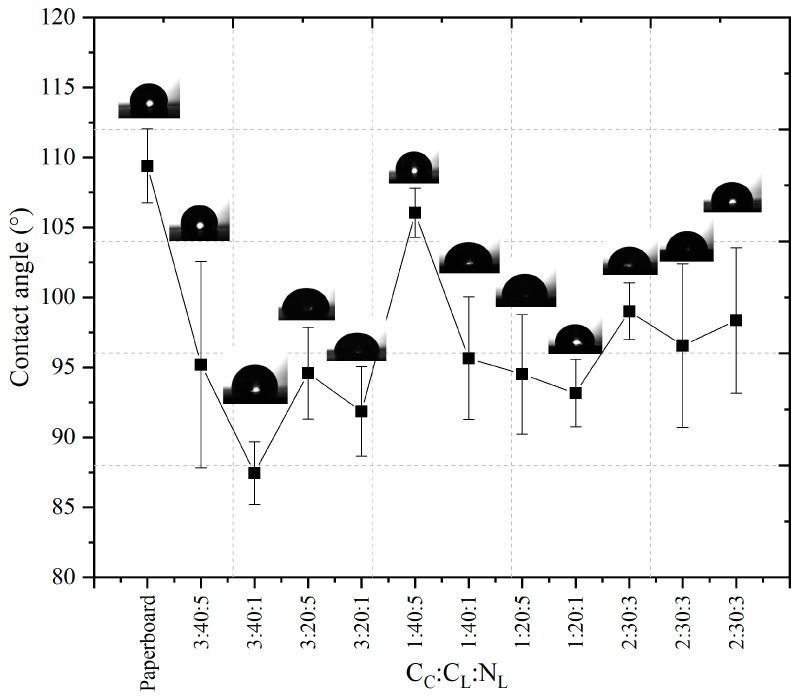
Contact angle between the water droplet and the solid surface of active film-paperboard systems.

Contact angle analysis is commonly used to determine surface wettability and is one of the most sensitive methods for chemical characterization of a material’s surface [36]. Some factors may influence the measurement’s accuracy, such as the temperature, type of liquid measured, drop size, surface heterogeneity, and surface roughness [37]. The CA values of the active film-paperboard systems were between hydrophobic (θ > 90°) and hydrophilic (θ < 90°) (Figure 3).

The uncoated paperboard (109.17 ± 1.53°) presented the highest contact angle value, indicating a more hydrophobic material than the active film-paperboard systems. The effect on the surface and heterogeneity created by hydrophobic domains and roughness retarded the wetting effect [38]. This may be attributed to the hydrophobic nature of empty air-containing pores, which may have hindered liquid absorption when a drop was thrown on the paper [39]. The lower values of the CA of the active coated paperboard could be associated with the hydrophilic nature of the chitosan coating. The paperboard coated with alginate and soy protein decreased the water droplets’ CA, possibly due to the formation of smoother and more homogeneous paperboard surfaces by filling the rough surfaces or pores with hydrophilic biopolymers [40]. The active film-paperboard systems in all treatments showed lower CA values, even with higher percentages of chitosan and lemongrass essential oil in the formulation. Cellulose fibers embedded in chitosan filled the pores of the papers and provided a hydrophilic character to the surfaces of the coated papers [9]. The CA achieved the highest value by reducing the percentage of chitosan and increasing the lemongrass essential oil concentration (1:40:5) (Figure 3). The major effect was from the lemongrass essential oil concentration, which increased the CA in the active film-paperboard systems. Coating paper with beeswax and chitosan promoted particle agglomeration, forming a more hydrophobic surface, but the CA showed no statistically significant difference [41].

### 3.3. Tensile Properties

**Figure 4:**
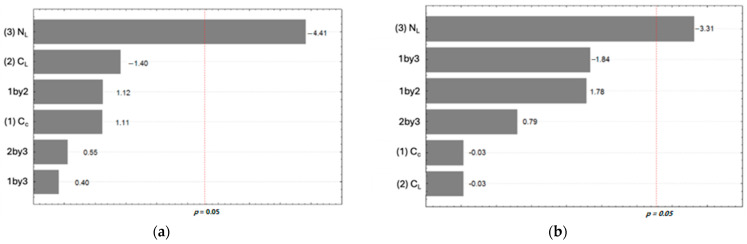
Independent variables’ effects (C_C_, C_L_, and N_L_) on the mechanical properties of active film-paperboard systems in the CD direction: (**a**) tensile strength and (**b**) elongation at break.

The active film-paperboard systems presented different mechanical tensile properties in the manufacture direction (MD) and cross-manufacture direction (CD) (Table 3). Increasing the NL from −1 (one layer) to +1 (five layers) promoted a significant reduction in tensile strength in the CD direction to the order of 3.06% (Figure 4).

For the MD direction, there was a significant positive effect from increasing the C_C_ from −1 (1%) to +1 (3%) (order of 1.09%), which also positively affected the interaction between the chitosan and coating layers (order of 0.64%). The cationic charge of chitosan could facilitate its adsorption onto cellulosic surfaces. The OH and NH_2_ groups on the chitosan molecule can participate in hydrogen bonding [42].

The N_L_ variable had a negative effect on the elongation at break. Increasing the N_L_ from −1 (one layer) to +1 (five layers) reduced the maximum force per unit width by 0.79%. The reduction in the elongation at break of the active film-paperboard systems was statistically significant only for the CD direction (Figure 4b).

### 3.4. Taber Stiffness

The active film-paperboard systems’ Taber stiffness (Rt) was modified compared with that of the uncoated paperboard (Table 3). The increase in C_C_ from −1 (1%) to +1 (3%) promoted a significant and positive effect on the Rt to the order of 0.98 mN. The increase in C_L_ from −1 (20%) to +1 (40%) promoted a significant and negative effect on the Rt, reducing it by 0.72 mN.

The chitosan coating on the paperboard surface increased the Taber stiffness compared with that of the uncoated paperboard. The chitosan (4%, *w*/*w*) coating kraft paper presented a higher stiffness in the longitudinal direction than the uncoated paper [43]. Chitosan has a possible plasticizing action, with reinforcement of the interfibrillar cellulose matrix [27]. Chitosan introduces a positive charge which interacts with the predominant negative charge of cellulose fibers, disturbing their interactions and cohesion [9].

### 3.5. Thermal Analysis: TG/DTG and DSC

**Figure 5:**
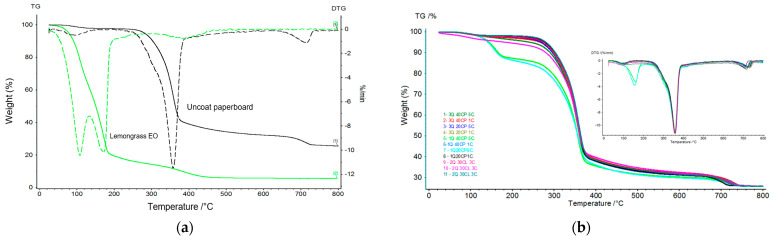

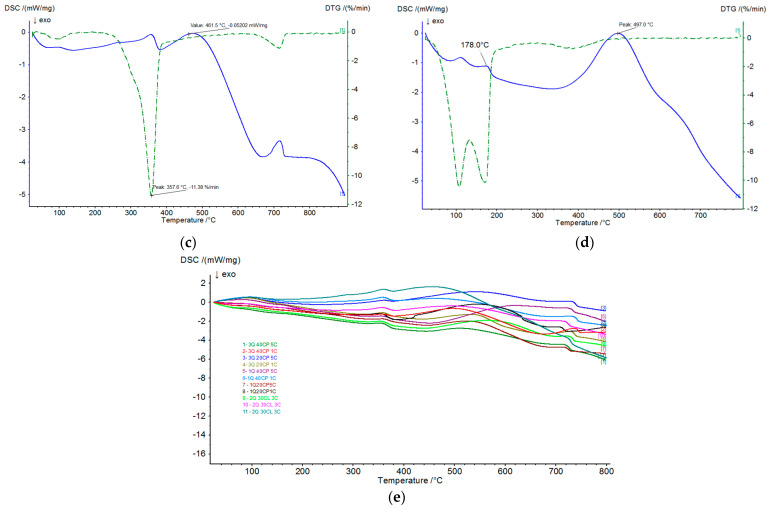
Thermograms: (**a**) TG/DTG of uncoated paperboard paper and lemongrass essential oil. (**b**) TG of active film-paperboard systems. (**c**) DSC of uncoated paperboard, (**d**) lemongrass essential oil, and (**e**) and active film-paperboard systems.

Thermogravimetric analyses are important for determining the thermal stability of polymers [44], temperature limits in an application [27], and components’ miscibility behavior. Figure 5 shows the weight loss as a function of the temperature variation for the lemongrass essential oil, uncoated paperboard (control), and active film-paperboard systems.

The TG/DTG curve of the lemongrass essential oil shows only one evaporation stage and quick evaporative weight loss from 107.6° C to 170.5 °C, exhibiting the maximum weight loss (82.4%). At 120 °C, the weight loss was 19.9% in the drying temperature of the active film-paperboard systems. The maximum weight loss can be attributed to the content of volatile compounds present in the essential oil. In this work, the lemongrass essential oil terpenoids comprised mainly citral content (88.1%), which corresponds to geranial (47.0%) and neral (41.11%). The weight loss began around room temperature, and evaporation ended at 166.7 °C in the TG/DTG profile revealed for lemongrass (*C. flexuosus*) [44].

The uncoated paperboard had a low initial weight loss attributed to removing the water absorbed by the cellulose fibers until 100 °C, and the weight loss was 51.6% at 327.8 °C. The cellulose samples studied in [45] showed a more pronounced weight loss degradation process around 280 °C. The main stage of decomposition occurs in the range of 240–370 °C when the cleavage of the cellulose glycosidic bonds reduces the degree of polymerization, leading to the formation of CO_2_, H_2_O, and an infinite amount of derived hydrocarbons. The paper started the decomposition at about 300 °C, and the peak was at 348 °C [46]. There are two instances of weight loss in chitosan; the first is at 50–150 °C, attributed to the evaporation of the water process, and the second is at 200–300 °C, due to degradation of the chitosan molecules [47].

The thermograms of active film-paperboard systems showed similar behavior, except for the 1:40:5 and 1:20:5 treatments. The multiple layers increased the essential oil concentration on the paperboard, affecting the material’s thermal stability and showing differences in initial weight loss due to the higher concentration of the essential oil. Chitosan presents depolymerization and pyrolytic decomposition (282–358 °C) [47]. This range of degradation overlaps with cellulose degradation. In the 300–400 °C range, major thermal degradation occurred in all active film-paperboard systems.

Endothermic peaks in the lemongrass essential oil sample close to 110.5 °C and 172.3 °C were related to the vaporization process of the volatiles, and the one at 497 °C was related to the decomposition reaction of non-volatile compounds in the essential oil. The DSC temperature related to vaporization occurs at 230 °C [44]. In the active materials, the thermal events showed typical first-order transitions associated with the samples’ mass loss. For the paperboard, the DSC curve shows an endothermic peak at 96.1 °C relative to vaporization and other endothermic peaks at 355.2 °C, 471.8 °C, and 715.4 °C related to material decomposition. The active materials essentially showed the characteristics of the endothermic peaks of the paperboard.

### 3.6. FTIR-ATR Spectra

**Figure 6:**
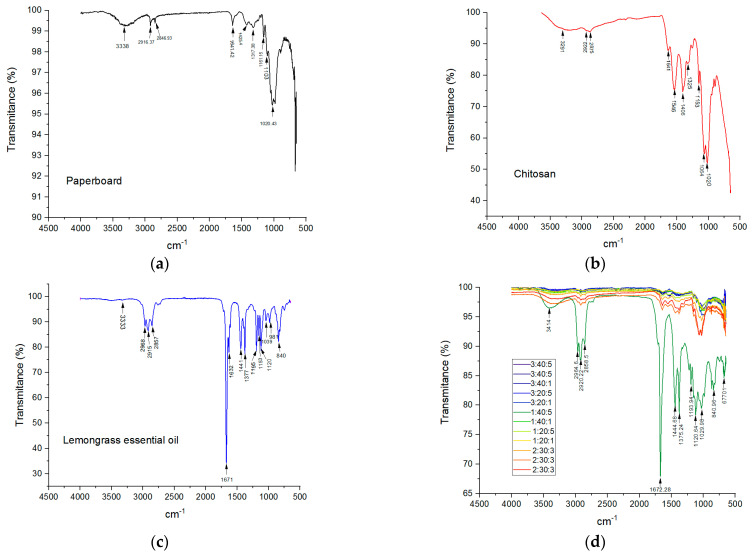
FTIR of the (**a**) paperboard, (**b**) chitosan, (**c**) lemongrass essential oil, and (**d**) active film-paperboard systems.

The vibration absorption bands, shown in Figure 6, provide information on the chemical species present in the active film-paperboard systems, allowing analysis of the structure and the interaction between the paperboard, chitosan, and lemongrass essential oil.

Among all treatments, the FTIR spectra of 1:40:5 showed that the chitosan and essential oil were shifted, indicating an electrostatic interaction between the anions in the oil molecules and the NH_3_^+^ of chitosan. The changes occurred in various peaks when chitosan was blended with the essential oil. Table 4 shows the spectra of the FTIR-ATR interpretation.

The spectra of active film-paperboard systems revealed that the absorption peaks dominated by chitosan, lemongrass essential oil, and paperboard decreased, confirming the incorporation of lemongrass essential oil into chitosan during the formation of coating layers on the paperboard.

### 3.7. X-Ray Diffraction (XRD)

**Figure 7:**
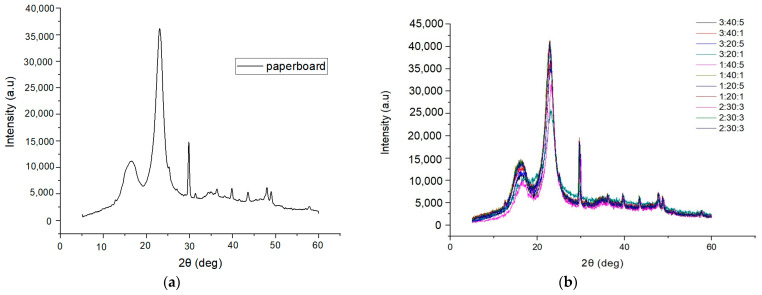
Diffractograms: (**a**) paperboard and (**b**) active materials coated with chitosan and lemongrass essential oil.

Figure 7 shows the X-ray diffraction behavior of the control (uncoated paperboard). In polymers, a marked amorphous halo can be observed in addition to crystalline domains, which are the ordered regions in the sample.

The diffractogram showed three well-defined peaks at approximately 2θ = 16.5°, 22.9° (highest peak), and 29.8° and an amorphous halo at 18.9°. The intensities of the crystalline peaks and the amorphous halo of the bleached cellulose of *Eucalyptus* spp. are characteristic of cellulose type I, in which the amorphous halo and the highest crystalline peak were located between the angles 18° ≤ 2θ ≤ 19° and 22° ≤ 2θ ≤ 23°, respectively [52]. The main diffraction signals on paper were 14.9° and 22.8°, angles usually attributed to the diffraction planes 101 and 002, respectively, which are proportional to type I cellulose [53].

There are two peaks in the diffraction pattern of chitosan [1]. The first is a reflection at 2θ = 11.4°, and another stronger reflection appears at 2θ = 20.1°, which also corresponds to the (100) reflection and the crystallinity, considering the degree of deacetylation of chitosan. Chitosan exhibits three reflection falls at 2θ = 11°, 20°, and 22°, showing quite broad lines, especially for the smaller diffraction angles, thereby indicating that long-range disorder is found in polymer samples [47]. The active film-paperboard systems’ diffractogram maintained the three well-defined peaks, and the crystallinity was between the corresponding values of chitosan and cellulose. This may be due to the reformation of hydrogen bonds between chitosan and cellulose during the essential oil’s dissolution and emulsion processes [54,55]. It was possible to observe in the 3:20:5 and 3:20:1 active film-paperboard systems a reduction in intensity to 25,000 u.a. for the peak near 2θ = 23°.

### 3.8. Cytotoxicity

Cytotoxicity analysis of the active material (1:40:5) was performed to investigate the safety of applying the coated material as primary packaging in direct contact with food. This is a new study in which the maximum limits for using lemongrass essential oil were not found in the literature, and the selection of the active material was based on the greater insecticidal efficiency of the active film-paperboard system [19].

The cytotoxicity analysis methodology adopted was based on the International Organization for Standardization’s ISO 10993-5 [33] and 10993-12 [34] standards for biological evaluation of devices for medical applications in direct contact with the skin or ingestion. Despite the high sensitivity, the method was an extrapolation for “in vitro” tests of the active material’s direct and indirect cell viability.

**Figure 8:**
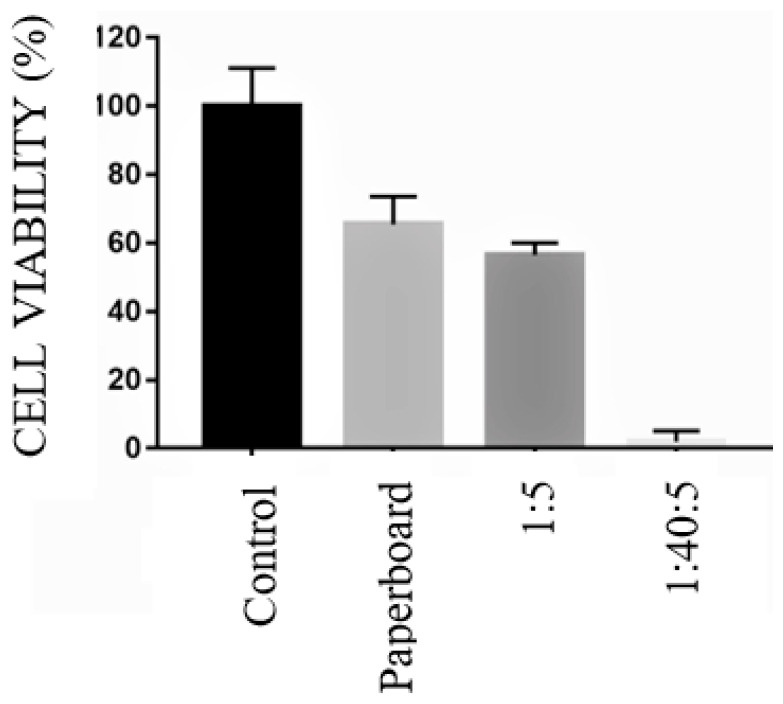
Direct form cytotoxicity results: control, uncoated paperboard, 1:5 (C_C_:N_L_), and 1:40:5 (C_C_:C_L_:N_L_).

The uncoated paperboard and paperboard coated with 1% chitosan (*w*/*v*) applied in five layers (1:5) were used as control materials. The analysis was performed in two ways: direct and indirect. The direct way consisted of directly applying discs of paperboard samples with and without the active coating in contact with the culture medium and cells (Figure 8).

The 1:40:5 active material did not show cell viability in the direct analysis, which was associated with the lemongrass essential oil. The uncoated paperboard and paperboard coated with chitosan (1:5) showed cell viability.

**Figure 9:**
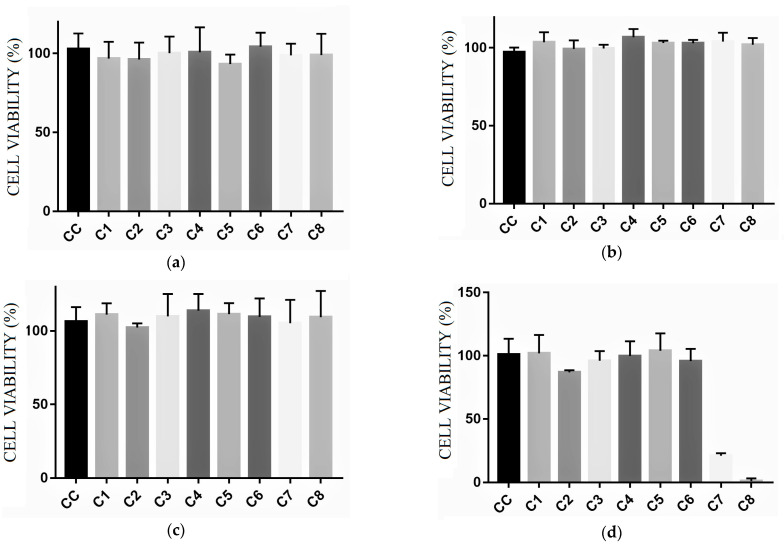
Indirect form cytotoxicity results: (**a**) control, (**b**) uncoated paperboard, (**c**) 1:5 (C_C_:N_L_), and (**d**) 1:40:5 (C_C_:C_L_:N_L_).

In the indirect form, extracts were prepared with the active coating materials from paperboard with and without the coating, using propylene glycol to extract all of the active compounds. The extract dilutions (C1–C8) were inoculated into the culture medium and the cells. Propylene glycol was prepared with a culture medium and cells as a CC cell control. The indirect cytotoxicity results can be analyzed in Figure 9.

The uncoated and coated with chitosan (1:5) paperboard showed cell viability. The 1:40:5 active material showed cell viability (80%) at concentrations equal to or lower than C6 (18.75 mg/mL). It is important to emphasize that the indirect methodology used is a drastic condition in which the samples were immersed in the solution to extract active compounds. This situation would not occur in the case of a material used as packaging for food farinaceous products.

The amount of lemongrass essential oil can be reduced in future works since selecting the 1:40:5 active material was based on the best performance concerning the insecticide action [26]. It was chosen for greater effectiveness against insect infestation. The coated material within the conditions worked, as it was not found in the literature’s concentration limits for lemongrass essential oil and is an innovative material.

Lemongrass essential oil is an active ingredient which is eligible for the use of minimal-risk pesticides [56]. In Title 21 Subchapter b, the FDA considers lemongrass oil as food for human consumption; in Part 182, it lists the substances as generally recognized as safe (GRAS); and in Section 182.20 for essential oils, it cites the EO of grass-lemon *C. citratus* as safe to be consumed by humans and does not establish maximum tolerance limits for this compound [57]. There is no tolerance or upper limit for using lemongrass essential oil. Lemongrass oil is classified as a food in the herbal category and, as such, is implicitly exempt from the tolerance requirement, representing a minimal risk to human health and the environment.

Other scientific works have applied lemongrass essential oil as an antimicrobial agent [58] and food preservative for control post-harvest and combined pathogenic fungi with chitosan [59]. The essential oil in the vapor phase is an effective antimicrobial and has advantages for developing new natural food preservatives [60].

The cytotoxicity results suggest a reduction in lemongrass essential oil within the cell viability range, which can reduce the insecticidal action of the active packaging system; however, they would still guarantee the death of *S. zeamais* insects [19]. It should be noted that in real conditions, this active packaging system would not be in a liquid condition and would not be ingested, and the probability of the volatile compounds of the lemongrass essential oil migrating to the food and causing cytotoxicity in humans and animals is extremely low (i.e., almost null) since the essential oil is classified as GRAS.

## 4. Conclusions

Active film-paperboard packaging systems were obtained from paperboard coatings with different formulations of chitosan film and lemongrass essential oil, as defined by the experimental design, presenting a yellowish color. The number of coating layers significantly influenced the mechanical properties (TS and Єr). By increasing the chitosan concentration, a stiffer material was formed in the longitudinal direction and reduced in the transverse direction. The mechanical resistance should be considered to depend on the performance of the packaging application proposal. The active material presented the characteristics of the chemical species of chitosan, cellulose, and lemongrass essential oil. The active film-paperboard materials showed similar thermal loss behaviors, except for the tests on 1:40:5 and 1:20:5 with a higher C_L_. The thermal events of the active materials were first-order transitions related to the sample mass loss. The systems presented three characteristic peaks in the X-ray diffraction analysis—2θ = 16.5°, 22.9°, and 29.8°—and an amorphous halo at 18.9°. The developed material is a sustainable and promising packaging alternative for reducing the use of chemical pesticides. Reducing the lemongrass concentration in the coating formulation is interesting for maintaining cell viability. This active material enables recycling and biodegradation, facilitated by packaging produced using natural polymers from renewable sources (chitosan and cellulose). The material developed is a sustainable and promising packaging alternative for reducing the use of chemical pesticides in grains with a high incidence of insect attacks, such as wheat grain and grain-derived food products.

## Figures and Tables

**Table 1:** Experimental Design 2^3^. Levels and concentration (*w*/*v* %) of chitosan (C_C_) and lemongrass essential oil (C_L_), respectively, and the number of layers (N_L_) in active paper-film system denomination.

Test	Levels Coded (Real Values)			Formulation (C_C_:C_L_:N_L_)
	C_C_	C_L_	N_L_	
1	1 (3%)	1 (40%)	1 (5N_L_)	3:40:5
2	1 (3%)	1 (40%)	−1 (1N_L_)	3:40:1
3	1 (3%)	−1 (20%)	1 (5N_L_)	3:20:5
4	1 (3%)	−1 (20%)	−1 (1N_L_)	3:20:1
5	−1 (1%)	1 (40%)	1 (5N_L_)	1:40:5
6	−1 (1%)	1 (40%)	−1 (1N_L_)	1:40:1
7	−1 (1%)	−1 (20%)	1 (5N_L_)	1:20:5
8	−1 (1%)	−1 (20%)	−1 (1N_L_)	1:20:1
9	0 (2%)	0 (30%)	0 (3N_L_)	2:30:3
10	0 (2%)	0 (30%)	0 (3N_L_)	2:30:3
11	0 (2%)	0 (30%)	0 (3N_L_)	2:30:3

**Table 2:** Color parameter values (L*, a*, and b*) on the coating surface of active film-paper systems with chitosan and lemongrass essential oil.

Test	Formulation (C_C_:C_L_:N_L_)	L*	a*	b*	Hab (°)
1	3:40:5	86.93 ± 1.03	0.68 ± 0.18	21.42 ± 1.35	88.21 ± 0.41
2	3:40:1	83.76 ± 0.48	1.95 ± 0.29	15.47 ± 1.11	82.78 ± 1.14
3	3:20:5	83.08 ± 1.62	0.30 ± 0.24	25.11 ± 3.91	89.36 ± 0.48
4	3:20:1	80.69 ± 0.86	2.75 ± 0.17	15.26 ± 0.51	79.78 ± 0.33
5	1:40:5	81.16 ± 2.19	2.16 ± 0.17	22.83 ± 1.12	84.56 ± 0.48
6	1:40:1	82.88 ± 0.55	2.91 ± 0.11	15.04 ± 0.55	79.04 ± 0.09
7	1:20:5	82.69 ± 0.84	1.72 ± 0.16	16.19 ± 0.66	83.94 ± 0.38
8	1:20:1	81.26 ± 0.26	3.16 ± 0.02	16.42 ± 0.11	79.12 ± 0.05
9	2:30:3	81.88 ± 1.30	1.90 ± 0.17	15.53 ± 0.48	83.05 ± 0.47
10	2:30:3	81.59 ± 0.70	1.83 ± 0.34	15.21 ± 0.35	83.46 ± 1.34
11	2:30:3	82.76 ± 0.46	1.95 ± 0.29	15.47 ± 1.11	82.78 ± 1.14

**Table 3:** Tensile strength (TS), elongation at break (Єr), and Taber rigidity (Rt) in manufacture direction (MD) and cross-manufacture direction (CD) of active film-paper systems using different chitosan and lemongrass essential oil formulations.

Test	Formulation (C_C_:C_L_:N_L_)	Average Thickness (µm)	TS (MPa)		Єr (%)		Rt (mN)	
			MD	CD	MD	CD	MD	CD
1	3:40:5	435 ± 3	26.14 ± 1.21	14.06 ± 0.61	2.16 ± 0.14	5.11 ± 0.25	15.67 ± 0.58	7.48 ± 0.17
2	3:40:1	394 ± 4	27.61 ± 1.30	15.15 ± 0.23	2.05 ± 0.16	5.60 ± 0.41	15.30 ± 0.31	6.96 ± 0.47
3	3:20:5	487 ± 2	25.02 ± 1.46	12.93 ± 1.10	2.21 ± 0.14	4.54 ± 0.33	17.16 ± 0.69	8.75 ± 0.40
4	3:20:1	399 ± 4	29.12 ± 2.16	15.65 ± 1.18	2.16 ± 0.21	5.63 ± 0.28	16.18 ± 0.58	7.60 ± 0.46
5	1:40:5	448 ± 2	23.38 ± 1.47	13.27 ± 0.65	1.95 ± 0.09	4.94 ± 0.26	15.23 ± 0.95	6.96 ± 0.40
6	1:40:1	399 ± 4	27.27 ± 1.26	16.40 ± 0.82	2.05 ± 0.13	5.23 ± 0.40	15.05 ± 0.73	7.33 ± 0.32
7	1:20:5	423 ± 4	25.68 ± 1.48	15.47 ± 0.78	2.18 ± 0.13	5.28 ± 0.25	15.54 ± 0.75	7.60 ± 0.40
8	1:20:1	385 ± 2	28.48 ± 1.04	17.09 ± 0.63	2.12 ± 0.12	5.44 ± 0.26	14.56 ± 0.45	7.67 ± 0.33
9	2:30:3	426 ± 4	26.46 ± 1.06	13.93 ± 0.12	2.21 ± 0.10	5.41 ± 0.24	16.28 ± 0.47	7.58 ± 0.54
10	2:30:3	424 ± 4	26.01 ± 1.02	14.36 ± 0.76	2.22 ± 0.12	5.00 ± 0.47	16.28 ± 0.80	7.60 ± 0.52
11	2:30:3	429 ± 3	25.10 ± 0.90	14.00 ± 1.09	2.19 ± 0.14	5.03 ± 0.36	15.79 ± 0.87	8.12 ± 0.76

**Table 4:** Interpretation of FTIR-ATR.

Component	Wave Number (cm^−1^)	Interpretation
Cellulose [48]	3338 2916, 2846 1161, 1103 1425 1020	O-H stretching C-H stretching C-O-C stretching HCH and OCH in-plane bending vibration C–C, C–OH, and C–H ring and side group vibrations
Chitosan [49]	3291 2922 2875 1641 1325 1546 1153 1064, 1020	N-H and O-H stretching and intramolecular hydrogen bond stretching C-H symmetric stretching C-H asymmetric stretching C=O stretching of amide I (presence of residual N-acetyl groups) C-N stretching of amide III (presence of residual N-acetyl groups) N-H bending of amide II asymmetric stretching of the C-O-C bridge C-O stretching
Lemongrass essential oil [50,51]	2968 2915 and 2857 1671 1632 1441 1377 1195–1120 840	predominant asymmetric stretching of -CH3 corresponding to an alkyl saturated aliphatic group -CH2 symmetric and asymmetric stretching C=C vibrations (cis and trans), confirming the presence of conjugated double bonds (C=C-CHO) in citral stretching of C=O of the aldehyde group -CH2 bending -CH3 bending stretching of -C-O and vibrations of the -CH skeleton CH=CH trans unsaturation and 1, 3 disubstitution or 1, 4 disubstitution were also observed

## Data Availability

The raw data supporting the conclusions of this article will be made available by the authors on request.

## References

[1] Tian B., Liu J., Yang W., Wan J.B. (2023). Biopolymer Food Packaging Films Incorporated with Essential Oils. J. Agric. Food Chem..

[2] Koketso Ncube L., Ude A.U., Nifise Ogunmuyiwa E., Zulkifli R., Beas I.N. (2021). An Overview of Plastic Waste Generation and Management in Food Packaging Industries. Recycling.

[3] Din M.I., Ghaffar T., Najeeb J., Hussain Z., Khalid R., Zahid H. (2020). Potential Perspectives of Biodegradable Plastics for Food Packaging Application-Review of Properties and Recent Developments. Food Addit. Contam. Part A Chem. Anal. Control Expo. Risk Assess..

[4] Pan Y., Farmahini-farahani M., Hearn P.O., Xiao H., Ocampo H. (2016). An Overview of Bio-Based Polymers for Packaging Materials. J. Bioresour. Bioprod..

[5] Song Z., Tang J., Li J., Xiao H. (2013). Plasma-Induced Polymerization for Enhancing Paper Hydrophobicity. Carbohydr. Polym..

[6] Adibi A., Trinh B.M., Mekonnen T.H. (2023). Recent Progress in Sustainable Barrier Paper Coating for Food Packaging Applications. Prog. Org. Coat..

[7] Poulose S., Toriseva J., Lahti J., Jönkkäri I., Hedenqvist M.S., Kuusipalo J. (2022). A Green High Barrier Solution for Paperboard Packaging Based on Potato Fruit Juice, Poly(Lactic Acid), and Poly(Butylene Adipate Terephthalate). ACS Appl. Polym. Mater..

[8] Kansal D., Rabnawaz M. (2021). Fabrication of Oil- and Water-Resistant Paper without Creating Microplastics on Disposal. J. Appl. Polym. Sci..

[9] Bordenave N., Grelier S., Pichavant F., Coma V. (2007). Water and Moisture Susceptibility of Chitosan and Paper-Based Materials: Structure-Property Relationships. J. Agric. Food Chem..

[10] Srinivasa P.C., Ramesh M.N., Kumar K.R., Tharanathan R.N. (2003). Properties and Sorption Studies of Chitosan—Polyvinyl Alcohol Blend Films. Carbohydr. Polym..

[11] Kumar M.N.V.R. (2000). A Review of Chitin and Chitosan Applications. React. Funct. Polym..

[12] Grinfelds U., Zoldners J., Passas R., Vikele L. (2017). Effect of chitosan on properties of paper for packaging. Cellul. Chem. Technol..

[13] Kjellgren H., Ga M. (2006). Barrier and Surface Properties of Chitosan-Coated Greaseproof Paper. Carbohydr. Polym..

[14] Khwaldia K., Basta A.H., Aloui H., El-Saied H. (2014). Chitosan-Caseinate Bilayer Coatings for Paper Packaging Materials. Carbohydr. Polym..

[15] Yoshida C.M.P., Oliveira E.N., Franco T.T. (2009). Chitosan Tailor-Made Films: The Effects of Additives on Barrier and Mechanical Properties. Packag. Technol. Sci..

[16] Tanpichai S., Srimarut Y., Woraprayote W., Malila Y. (2022). Chitosan Coating for the Preparation of Multilayer Coated Paper for Food-Contact Packaging: Wettability, Mechanical Properties, and Overall Migration. Int. J. Biol. Macromol..

[17] Jumaa M., Mu B.W. (1999). Physicochemical Properties of Chitosan-Lipid Emulsions and Their Stability during the Autoclaving Process. Int. J. Pharm..

[18] Yildirim S., Röcker B., Pettersen M.K., Nilsen-Nygaard J., Ayhan Z., Rutkaite R., Radusin T., Suminska P., Marcos B., Coma V. (2017). Active Packaging Applications for Food. Compr. Rev. Food Sci. Food Saf..

[19] de Fátima Silva M., Maciel V.B.V., Noletto A.P.R., Venturini A.C., de Carvalho R.A., Yoshida C.M.P. (2022). Chitosan Active Coating on Paperboard Surface Forming an Anti-Insect Grain-Based Food Packaging. Packag. Technol. Sci..

[20] Djebbi T., Ascrizzi R., Bedini S., Farina P., Sanmartin C., Mediouni Ben Jemâa J., Bozzini M.F., Flamini G., Conti B. (2024). Physicochemical and Repellent Properties of Chitosan Films Loaded with Essential Oils for Producing an Active Packaging Effective Against the Food Pest *Sitophilus oryzae*. J. Stored Prod. Res..

[21] Negrelle R.R.B., Gomes E.C. (2007). *Cymbopogon citratus* (DC.) Stapf: Chemical Composition and Biological Activities. Rev. Bras. De Plantas Med..

[22] Lawal O.A., Ogundajo A.L., Avoseh N.O., Ogunwande I.A. (2017). Cymbopogon citratus. Medicinal Spices and Vegetables from Africa: Therapeutic Potential Against Metabolic, Inflammatory, Infectious and Systemic Diseases.

[23] Saddiq A.A., Khayyat S.A. (2010). Chemical and Antimicrobial Studies of Monoterpene: Citral. Pestic. Biochem. Physiol..

[24] Zhang W., Xiao H., Qian L. (2014). Beeswax-Chitosan Emulsion Coated Paper with Enhanced Water Vapor Barrier Efficiency. Appl. Surf. Sci..

[25] Despond S., Espuche E., Cartier N., Domard A. (2005). Barrier Properties of Paper-Chitosan and Paper-Chitosan-Carnauba Wax Films. J. Appl. Polym. Sci..

[26] ASTM (2010). Annual Book of ASTM Standards.

[27] Yoshida C.M.P., Maciel V.B.V., Mendonça M.E.D., Franco T.T. (2014). Chitosan Biobased and Intelligent Films: Monitoring PH Variations. LWT-Food Sci. Technol..

[28] (2003). Annual Book of ASTM Standards. Standard Test Method for Surface Wettability of Paper (Angle-of-Contact Method).

[29] Rabe A., Aliero B., Galadima A., Baqi A. (2018). Thermal Decomposition of Camel Grass and Lemon Grass. J. Sci. Res. Rep..

[30] Kriegner D., Matěj Z., Kužel R., Holý V. (2015). Powder Diffraction in Bragg-Brentano Geometry with Straight Linear Detectors. J. Appl. Crystallogr..

[31] (2016). Annual Book of ASTM Standards. Standard Test Method for Tensile Properties of Paper and Paperboard Using Constant-Rate-of-Elongation Apparatus.

[32] (2007). Annual Book of ASTM Standards. Standard Test Method for Resistence to Bending of Paper Paperboard (Taber-Type Tester in Basic Configuration).

[33] (2009). Biological Evaluation of Medical Devices—Tests for In Vitro Cytotoxicity.

[34] (2021). Biological Evaluation of Medical Devices-Part 12: Sample Preparation and Reference Materials Évaluation Biologique Des Dispositifs Médicaux-Partie 12: Préparation Des Échantillons et Matériaux de Référence Copyright Protected Document.

[35] Gällstedt B.M., Brottman A., Hedenqvist M.S. (2005). Packaging-Related Properties of Protein- and Chitosan-Coated Paper and Science. Packag. Technol. Sci. Int. J..

[36] Stepien M., Saarinen J.J., Teisala H., Tuominen M., Aromaa M., Kuusipalo J., Mäkelä J.M., Toivakka M. (2012). Surface Chemical Characterization of Nanoparticle Coated Paperboard. Appl. Surf. Sci..

[37] Kannangara D., Shen W. (2008). Roughness Effects of Cellulose and Paper Substrates on Water Drop Impact and Recoil. Colloids Surf. A Physicochem. Eng. Asp..

[38] Modaressi H., Garnier G. (2002). Mechanism of Wetting and Absorption of Water Droplets on Sized Paper: Effects of Chemical and Physical Heterogeneity. Langmuir.

[39] Guillaume C., Pinte J., Gontard N., Gastaldi E. (2010). Wheat Gluten-Coated Papers for Bio-Based Food Packaging: Structure, Surface and Transfer Properties. Food Res. Int..

[40] Rhim J., Lee J., Hong S. (2006). Water Resistance and Mechanical Properties of Biopolymer (Alginate and Soy Protein) Coated Paperboards. LWT-Food Sci. Technol..

[41] Gal M.R., Rahmaninia M., Hubbe M.A. (2023). A Comprehensive Review of Chitosan Applications in Paper Science and Technologies. Carbohydr. Polym..

[42] Reis A.B., Yoshida C.M.P., Reis A.P.C., Franco T.T. (2011). Application of Chitosan Emulsion as a Coating on Kraft Paper. Polym. Int..

[43] Abdelrazek E.M., Elashmawi I.S., Labeeb S. (2010). Chitosan Filler Effects on the Experimental Characterization, Spectroscopic Investigation and Thermal Studies of PVA/PVP Blend Films. Physica B.

[44] Fazzio P., Martinez M., Benites C., Regina M., Maciel W. (2011). Thermal Characterization of Orange, Lemongrass, and Basil Essential Oils. Chem. Eng. Trans..

[45] Poletto M., Pistor V., Zattera A.J. (2013). Structural Characteristics and Thermal Properties of Native Cellulose. Cellul.–Fundam. Asp..

[46] Habibie S., Hamzah M., Anggaravidya M., Kalembang E. (2016). The Effect of Chitosan on Physical and Mechanical Properties of Paper. J. Chem. Eng. Mater. Sci..

[47] Tripathi S., Mehrotra G.K., Dutta P.K. (2010). Preparation and Physicochemical Evaluation of Chitosan/Poly(Vinyl Alcohol)/Pectin Ternary Film for Food-Packaging Applications. Carbohydr. Polym..

[48] Dai D., Fan M. (2011). Investigation of the Dislocation of Natural Fibres by Fourier-Transform Infrared Spectroscopy. Vib. Spectrosc..

[49] Mauricio-Sánchez R.A., Salazar R., Luna-Bárcenas J.G., Mendoza-Galván A. (2018). FTIR Spectroscopy Studies on the Spontaneous Neutralization of Chitosan Acetate Films by Moisture Conditioning. Vib. Spectrosc..

[50] Vazquez-Briones M.D.C., Hernandez L.R., Guerrero-Beltran J.A. (2015). Physicochemical and Antioxidant Properties of *Cymbopogon citratus* Essential Oil. J. Food Res..

[51] Wany A., Kumar A., Nallapeta S., Jha S., Nigam V.K., Pandey D.M. (2014). Extraction and Characterization of Essential Oil Components Based on Geraniol and Citronellol from Java Citronella (Cymbopogon Winterianus Jowitt). Plant Growth Regul..

[52] Cristina Lengowski E., Ines Bolzon de Muniz G., Nisgoski S., Luiz Esteves Magalhães W. (2013). Scientia ForeStaliS Avaliação de Métodos de Obtenção de Celulose Com Diferentes Graus de Cristalinidade (Cellulose Acquirement Evaluation Methods with Different Degrees of Crystallinity). Sci. For..

[53] Chen J., Han X., Fang Z., Cheng F., Zhao B., Lu P., Li J., Dai J., Lacey S., Elspas R. (2015). Rapid Dissolving-Debonding Strategy for Optically Transparent Paper Production. Sci. Rep..

[54] Lin S., Chen L., Huang L., Cao S., Luo X., Liu K. (2015). Novel Antimicrobial Chitosan-Cellulose Composite Films Bioconjugated with Silver Nanoparticles. Ind. Crops Prod..

[55] Xu Y.X., Kim K.M., Hanna M.A., Nag D. (2005). Chitosan-Starch Composite Film: Preparation and Characterization. Ind. Crops Prod..

[56] Baker B.P., Grant J.A. (2018). Active Ingredients Eligible for Minimum Risk Pesticide Use: Overview of the Profiles.

[57] FDA (2018). Code of Federal Regulations. Food and Drugs. Food for Human Consumption. Title 21.

[58] Boukhatem M.N., Ferhat M.A., Kameli A., Saidi F., Kebir H.T. (2014). Lemon Grass (*Cymbopogon citratus*) Essential Oil as a Potent Anti-Inflammatory and Antifungal Drugs. Libyan J. Med..

[59] Munhuweyi K., Caleb O.J., Lennox C.L., van Reenen A.J., Opara U.L. (2017). In Vitro and in Vivo Antifungal Activity of Chitosan-Essential Oils against Pomegranate Fruit Pathogens. Postharvest Biol. Technol..

[60] Hadjilouka A., Polychronopoulou M., Paramithiotis S., Tzamalis P., Drosinos E.H. (2015). Effect of Lemongrass Essential Oil Vapors on Microbial Dynamics and *Listeria Monocytogenes* Survival on Rocket and Melon Stored under Different Packaging Conditions and Temperatures. Microorganisms.

